# The relationship between mild cognitive impairment and postoperative delirium undergoing total knee arthroplasty: The PNDABLE study

**DOI:** 10.3389/fnagi.2022.959510

**Published:** 2022-09-29

**Authors:** Bin Wang, Chuanlin Mu, Xinhui Tang, Fei Wang, Gaofeng Zhang, Jiahan Wang, Rui Dong, Xu Lin, Yanlin Bi

**Affiliations:** ^1^Department of Anesthesiology, Qingdao Municipal Hospital Affiliated to Qingdao University, Qingdao, China; ^2^Department of Anesthesiology, Dalian Medical University, Dalian, China; ^3^Department of Anesthesiology, Nanjing Medical University, Nanjing, China; ^4^Department of Anesthesiology, Drum Tower Hospital Affiliated to Nanjing University Medical School, Nanjing, China

**Keywords:** mild cognitive impairment, biomarkers, neurodegeneration, geriatric, postoperative delirium

## Abstract

**Background:**

Patients undergoing surgery are at a higher risk of developing postoperative delirium (POD) as a result of anesthesia and surgical procedures. This study examined the association between POD and mild cognitive impairment (MCI) and whether MCI influences POD through the core pathology of POD.

**Methods:**

We enrolled Chinese Han patients undergoing unilateral total knee arthroplasty (aged 50–90, weighing 50–80 kg, and using ASAI-II), combined with epidural anesthesia between October 2020 and June 2021. All the participants were assessed using Winblad's criteria for diagnosing MCI on pre-operation and using the Confusion Assessment Method (CAM) and the Memorial Delirium Assessment Scale (MDAS) postoperative 1–7 days (or before discharge) for diagnosing POD by an anesthesiologist. Cerebrospinal fluid (CSF) biomarkers of POD were measured by enzyme-linked immunosorbent assay (ELISA). To examine the mechanism by which POD pathologies affect cognition, causal mediation analyses were performed.

**Results:**

POD incidence was 20.2%, including 32.5% in the MCI group and 12.4% in the non-mild cognitive impairment (NMCI) group. The MCI and CSF levels of T-tau and P-tau were risk factors, and the CSF levels of Aβ_42_, Aβ_42_/ T-tau, and Aβ_42_/ P-tau were protective factors in POD (*p* < 0.05). Part of the effects of MCI on cognition can be attributed to amyloid pathology and tau.

**Conclusion:**

MCI may be a reasonably good prognostic factor in POD development. Overall, amyloid pathology and tau protein might partially mediate the influence of MCI on POD.

**Clinical trial registration:**

www.clinicaltrials.gov, identifier: ChiCTR2000033439.

## Background

Postoperative delirium (POD) is a serious complication resulting from anesthesia and surgery (Safavynia and Goldstein, 2018). Patients suffering from POD experience temporary or permanent cognitive decline, deterioration in verbal comprehension, and difficulties adapting to social situations. POD primarily affects the aged (> 65 years old) (Ramaiah and Lam, 2009), and it leads to increased mortality rates and hospitalization lengths, in addition, other complications, such as Alzheimer's disease (AD) and consequent higher treatment expenses (Steinmetz et al., 2009). POD is common and of great clinical significance. However, the mechanisms behind POD remain largely unexplained with no reliable biomarkers have been identified to date.

Amyloid beta (Aβ), including Aβ_40_ and Aβ_42_, is a component of AD senile plaques. Abnormal accumulation of Aβ initiates the cascade reactions within nerve cells, such as synaptic damage, excessive phosphorylation of Tau protein, formation of neurofibrillary tangles, and eventual damage on neurons, resulting in neuronal death and memory and cognitive dysfunction (Plotkin and Cashman, 2020). The microtubule-associated protein Tau plays an important role in the structure and function of microtubules in neurons (Guo et al., 2017). Phosphorylated Tau protein does not bind to tubulin but inhibits and destroys the formation of microtubules and finally results in cognitive dysfunction (Li et al., 2007). A recent study has revealed that preoperative positive cerebrospinal fluid (CSF) Aβ and Tau may be associated with an increased risk of POD (Fong et al., 2021). In several studies of senior adults without a previous diagnosis of dementia, preoperative positive CSF Aβ, Tau, and phosphorylated Tau (P-tau) were indicated as the strongest independent predictors of POD after elective arthroplasty (Planel et al., 2007; Nakajima et al., 2015; Cunningham et al., 2019; Dutkiewicz et al., 2020). In the present, Aβ and P-tau have been widely accepted as contributing factors in the development of POD.

In clinical and laboratory settings, mild cognitive impairment (MCI), the transitional state between normal aging and dementia, is an essential diagnostic indicator. MCI is associated with elevated prevalence of dementia, and the progression rate varies from 10 to 15% annually (Wang et al., 2015; Xue et al., 2017). MCI has three specificities: some cases get worse and develop into AD; some cases remain at this transitional stage for a considerable time, while a small number of cases could resume normal cognitive ability after active treatment. A previous study has exhibited a decrease in the level of Aβ_42_ in CSF with the aggravation of patients with MCI (Fjell and Walhovd, 2012). Due to shared neuropathological mechanisms between AD and POD, MCI has been linked with neurodegeneration, suggesting a possible correlation between MCI and POD through changes in CSF Aβ.

Therefore, we planned to conduct a prospective, observational cohort study to investigate the relationship of MCI with POD and CSF POD biomarkers in addition to better understand the role of CSF POD biomarkers in mitigating the effects of MCI on POD. The aforementioned analyses were based on the PNDABLE (Perioperative Neurocognitive Disorder and Biomarker Lifestyle) study.

## Materials and methods

### PNDABLE study

The PNDABLE aims to investigate the pathogenesis, risk factors, and biomarkers of perioperative neurocognitive disorders (PND) in the Han Chinese population from northern China. It is deployed to identify lifestyle factors that may affect the risk of PND in the non-demented Han Chinese population from northern China to provide a foundation for disease prevention and early diagnosis. This study has been registered in the Chinese Clinical Trial Registry (clinical registration No. ChiCTR2000033439) and approved by the Ethics Committee of Qingdao Municipal Hospital. All the patients or their legal representatives signed written consent before CSF sample collections.

### Participants

The Han Chinese patients underwent unilateral total knee arthroplasty (no gender limitations, aged, 50~90; weight, 50–80 kg, ASA I~II) combined with epidural anesthesia at Qingdao Municipal Hospital from October 2020 to June 2021 were enrolled to the PNDABLE study. The exclusion criteria include: (1) the preoperative MMSE score <24 points; (2) Drug or psychotropic substance abuse, as well as long-term use of steroid drugs and hormone drugs; (3) preoperative III–IV hepatic encephalopathy; (4) Recent major surgery; (5) Severe visual and hearing impairments; (6) Abnormal coagulation function before surgery;(7) central nervous system infection, head trauma, multiple sclerosis, neurodegenerative diseases other than AD (e.g., epilepsy, Parkinson's disease), or other major neurological disorders; (8) major psychological disorders;(9) severe systemic diseases (e.g., malignant tumors) that may affect CSF or blood levels of AD biomarkers, including Aβ and tau; (10) family history of genetic diseases.

**Figure 1:**
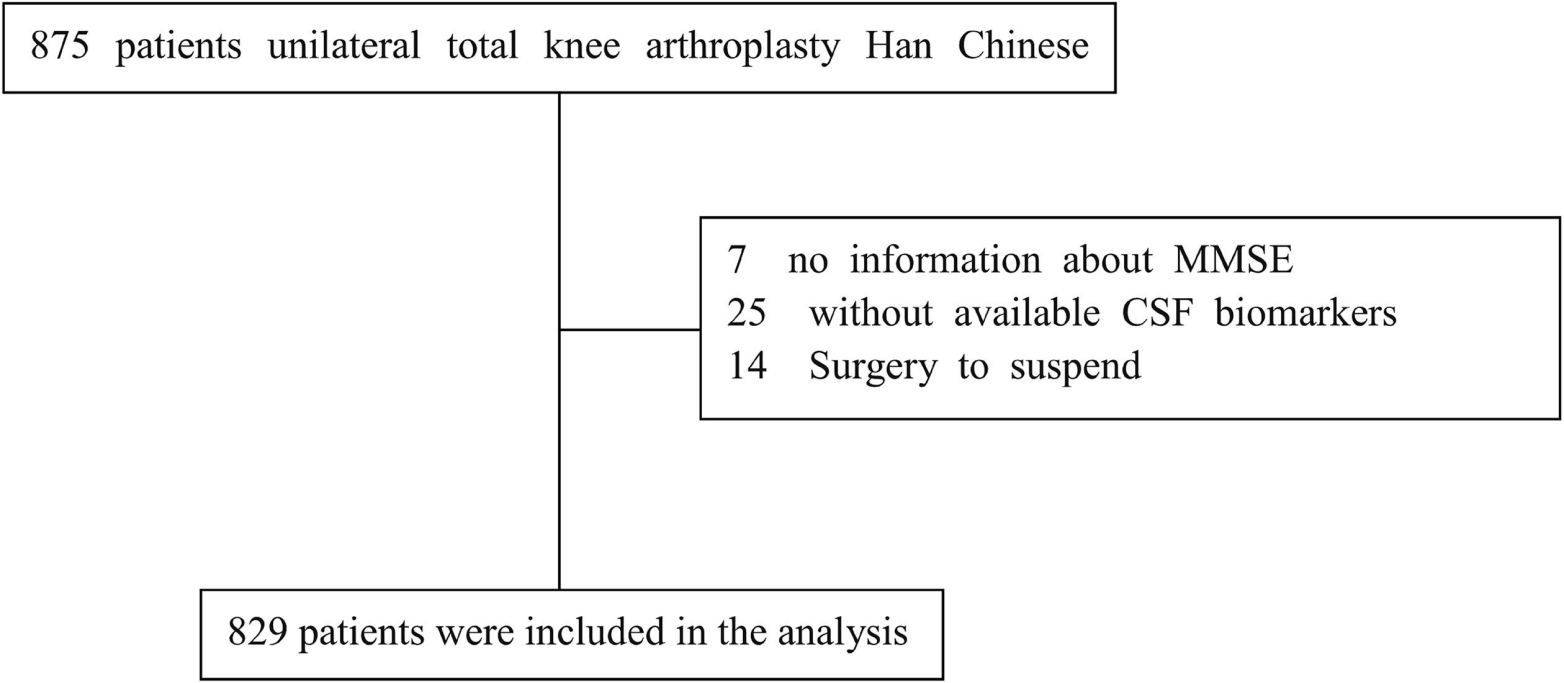
A flow diagram showed selection of eligible patients and the enrollment process.

A total of 875 cognitively normal participants with available covariates from PNDABLE were included. The participants were divided into two groups based on MCI occurrence: the MCI group and the NMCI group. A patient recruitment flowchart is shown in Figure 1.

The participants were prohibited from drinking for 6 h and eating for 8 h before surgery. After entering the operating room, the participants underwent combined spinal-epidural block, AS-E/SIIepidural, and a lumbar puncture kit was used, with the space between lumbar 3–4 spinous processes (L_3 − 4_) as the puncture point. With successful puncture, 2 ml of CSF was extracted from the subarachnoid space, followed by an injection of 2 to 2.5 ml of 0.66 percent ropivacaine (0.66%) in a duration of 30 s. During the surgery, to maintain blood pressure within +/– 20% of the baseline value, oxygen was inhaled by a mask at 5 L/min. A 5-mg injection of ephedrine was conducted intravenously in case intraoperative non-invasive blood pressure (NBP) was <90 mmHg (1 mmHg = 0.133 kPa) or reduced by over 20% of the baseline value. If the heart rate (HR) <50 beats/min, an injection of atropine 0.5 mg was given intravenously. Intravenous patient-controlled analgesia (butorphanol, 0.1 mg/ml + tropisetron, 50 g/ml, diluted with normal saline to a total volume of 100 ml) was used as acute postoperative pain management. The patients were sent to the anesthesia resuscitation room (PACU) after the operation.

The patients were interviewed the day before the surgery, in which their baseline information was collected, including age, gender, body mass index (BMI), ASA physical status, and educational background, etc. Other information about comorbidities and medical history was also gathered from the patients' medical records. As part of the procedure, the anesthesiologist compiled each patient's medical history, performed physical examination, and assessed each patient's cognitive abilities.

### CSF biomarkers of POD measurements

CSF samples were processed immediately within 2 h of standard lumbar puncture. The samples were centrifuged at 2,000 × g for 10 min, and then separated and stored in an enzyme-free EP (Eppendorf) tube (AXYGEN; PCR-02-C) at −80°C under the international BIOMARKAPD project for further use in the subsequent steps of this study.

CSF biomarkers of POD were measured by ELISA using the microplate reader (Thermo Scientific Multiskan MK3). CSF biomarkers of POD measurements were done with other ELISA kits [Aβ_42_ (BioVendor, Ghent, Belgium Lot: No. 296-64401), P-tau (BioVendor, Ghent, Belgium Lot: QY-PF9092), and T-tau (BioVendor, Ghent, Belgium Lot: No. EK-H12242)]. All ELISA measurements were performed by experienced technicians in strict accordance with the manufacturer's instructions. Technicians were blinded to the clinical information. The samples and standards were measured in duplicate, which was also applied in the statistical analyses. In addition to the CSF sample, each plate also included blank and internal control samples. In case deviation occurred on the quality control sample, the plate would be reanalyzed. Standards and CSF samples were analyzed with duplicate specimens, and the mean value of both specimens was used for subsequent statistical analysis. All the antibodies and plates were from a single lot to exclude variability between batches. Moreover, the within-batch CV was <5%, and the inter-batch CV was <15%.

### Diagnostic criteria for MCI

Winblad's criteria (Winblad et al., 2004) are applied to diagnose MCI. In this study, based on age and education-adjusted normative data, cognitive decline is defined as a deficit of minimum 1.5 SDs from premorbid performance on objective neuropsychological tests. The diagnosis requires both subjective and objective declines in cognition while retaining general functionality (i.e., only minimal change, if any, in basic and complex activities of daily living).

### Neuropsychological tests

The preoperative cognitive status of the participants was assessed by neurologists using the Mini-Mental State Examination (MMSE). The participants with an MMSE score <24 points were excluded.

After the surgery, an anesthesiologist performed delirium assessments at 9:00–10:00 a.m. and 2:00–3:00 p.m. two times daily for 1–7 days (till the participant was discharged). Pain was assessed by the numerical rating scale (NRS) of 0–10 (lower scores indicate lower levels of pain) (Leung et al., 2008). POD definition is in accordance with the Confusion Assessment Method (CAM) (Chung et al., 2016), and severity of POD is measured by the Memorial Delirium Assessment Scale (MDAS) (Inouye et al., 1990). Modified Telephone Interview for Cognitive Status (TICS-m) was administered to assess cognitive function after 6 months. The quality of life was evaluated with the World Health Organization Quality of Life brief version (WHOQOLBREF).

### Statistical analysis

The preliminary test in this study found that seven covariates were expected to enter the Logistic regression, the POD incidence was 10%, and the loss of follow-up rate was assumed to be 20%, so the required sample size was calculated to be 875 cases.

The Kolmogorov–Smirnov test is used to determine whether the measurement data conformed to normal distribution. Data conformed to normal distribution are expressed by mean ± standard deviation (SD), while the median (p25, p75) or a number (%) to express the data. The chi-square test (for categorical variables) and the Mann–Whitney U test (for continuous variables) are utilized to test the difference of baseline characteristics between the MCI and NMCI groups.

The first step was to evaluate the relationship between POD and MCI/CSF POD biomarkers. We used binary logistic regression analysis to analyze the association between POD and either MCI or CSF POD biomarkers. Additionally, we tested the relationships of MCI and CSF POD biomarkers, with consequent binary logistic regression analyses of MCI and CSF POD biomarkers performed. A two-factor ANOVA was deployed to determine whether gender affects the relationship between MCI and CSF POD biomarkers.

Second, sensitivity analyses were performed by adding more covariates, including BMI, type 2 diabetes (yes or no), hypertension (yes or no), smoking (yes or no), coronary heart disease (yes or no), and alcohol intake (yes or no) into multivariate logistic regression analysis.

Finally, an examination was conducted to determine whether the association between MCI and POD was mediated by CSF POD biomarkers by Stata MP 16.0 (Solvusoft Corporation, Inc., Chicago, Illinois, USA). Logistic regression models were fitted based on the methods. The first equation relates the mediator (CSF POD biomarkers) on the independent variable (MCI). The second equation relates the dependent variable (POD) on the independent variable (MCI). The third equation relates the dependent variable on both the independent variable and the mediator variable. Furthermore, the attenuation or indirect effect was also estimated, with the significance determined using 10,000 bootstrap iterations, where each path of the model was controlled for age, gender, years of education, and the MMSE score.

R software version 4.4.1 (R Foundation for Statistical Computing, Vienna, Austria) and GraphPad Prism version 7.00 (GraphPad Software, San Diego, CA) were used for statistical analyses and figure preparation. *P* < 0.05 was considered significant unless specifically noted.

## Results

### Comparison of demographic and clinical data of included participants

We found the incidence of POD is 20.2% (*n* = 168 of the 829 participants), respectively, 32.5% (105 of the 323 patients) in the MCI group and 12.4% (63 of the 506 participants) in the NMCI group (*p* < 0.05).

**Figure 2:**
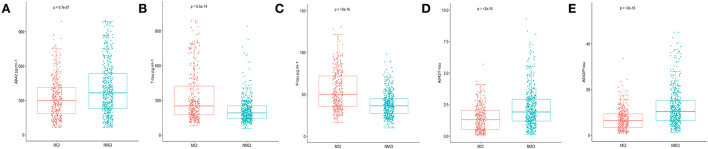
Distribution of CSF biomarkers levels for participants with and without MCI. **(A)** The concentrations of amyloid β_42_ (Aβ_42_) decreased in MCI patients compared with NNMCI patients. **(B)** Total-tau (T-tau) increased in MCI patients compared with NNMCI patients. **(C)** Phosphorylated total-tau (P-tau) increased in MCI patients compared with NNMCI patients. **(D)** Amyloid β_42_/ Total-tau (Aβ_42_/ T-tau) decreased in MCI patients compared with NNMCI patients. **(E)** Amyloid β_42_/ phosphorylated total-tau (Aβ_42_/ P-tau) decreased in MCI patients compared with NNMCI patients. *P*-values were assessed by Student's *T*-test.

In this study, the preoperative MMSE score showed no significant difference between the MCI group and the NMCI group. It is also unveiled that the participants in the MCI group demonstrate no cognitive function differences in the 6-month period. The demographic and clinical data of the participants are summarized, as shown in Table 1. The differences in CSF levels of Aβ_42_, T-tau, P-tau Aβ_42_/T-tau, and Aβ_42_/P-tau of the MCI group are statistically significant compared with the NMCI group (*p* < 0.05), as shown in Figure 2 and Table 2.

**Table 1:** Comparison of demographic and clinical data of unilateral total knee arthroplasty patients.

**Variable**	**MCI (*N* = 323)**	**NMCI (*N* = 506)**	***P*-value**
Age (year)	65.98 ± 9.34	60.37 ± 9.02	0.001
Sex (female/male)	182/141	317/189	0.071
Body mass index (kg.m^−2^)	25.30 ± 4.17	25.35 ± 3.92	0.827
Education level (year)	10.19 ± 3.89	10.49 ± 3.72	0.258
ASA physical status (I/II)	222/101	341/165	0.687
Alcohol abuse, *n* (%)	105 (32.5)	175 (34.6)	0.537
Hypertension, *n* (%)	105(32.5)	184(36.4)	0.255
Dependence on smoking, *n* (%)	80 (24.8)	151 (29.8)	0.112
Coronary heart disease, *n* (%)	45 (13.9)	50 (9.88)	0.334
Diabetes, *n* (%)	55 (14.8)	82 (10.8)	0.755
Duration of anesthesia (min)	145.11 ± 14.56	144.57 ± 14.98	0.607
Duration of surgery (min)	121.28 ± 12.17	121.32 ± 12.08	0.964
Estimated volume of infusion (ml)	844.43 ± 92.07	849.51 ± 92.94	0.442
Estimated blood loss (ml)	121.16 ± 13.01	121.32 ± 13.41	0.827
Preoperative MMSE scores	28 (27, 29)	28 (27, 30)	0.300
Postoperative the highest MDAS score	9 (8, 16)	1 (1, 2)	<0.001
Postoperative the highest NRS score	3 (2–3)	3 (2–3)	0.482
Postoperative delirium, *n* (%)	105 (32.5)	63 (12.4)	<0.001
TICS-m score	36.84 ± 3.16	36.73 ± 3.11	0.619
**WHOQOL-BREF score**
Physical domain	68.88 ± 3.56	69.08 ± 3.53	0.438
Psychological domain	75.45 ± 2.74	75.49 ± 2.75	0.830
Social relationships domain	67.41 ± 2.85	67.33 ± 2.89	0.727
Environment domain	83.20 ± 2.74	83.27 ± 2.76	0.705

POD, postoperative delirium; MMSE, mini-mental state examination; ASA, American Society of Anesthesiologists; MDAS, memorial delirium assessment scale; NRS, Numerical rating scale; SD, standard deviation; TICS-m, Telephone Interview for Cognitive Status; WHOQOL-BREF, World Health Organization Quality of Life -brief version.

**Table 2:** Comparison of CSF biomarkers of unilateral total knee arthroplasty patients.

**Variable**	**MCI (*N* = 323)**	**NMCI (*N* =506)**	***P*-value**
Preoperative CSFAβ_42_ (pg/ml)	297.97 (183.75, 414.86)	364.53 (230.28, 534.03)	<0.001
Preoperative CSF T-tau (pg/ml)	250.73 (172.93, 423.06)	190.59 (143.04, 251.49)	<0.001
Preoperative CSF P-tau (pg/ml)	49.70 (35.75, 72.01)	36.30 (27.36, 44.69)	<0.001
Preoperative CSFAβ_42_/ T-tau	1.29 (0.54, 2.03)	1.90 (1.19, 2.93)	<0.001
Preoperative CSFAβ_42_/ P-tau	6.30 (3.45, 9.40)	10.22 (6.30, 15.15)	<0.001

### Logistic regression analysis of the influencing factors of POD

In this study, logistic regression analysis results show that the participants with MCI are more prone to POD. MCI and CSF levels of T-tau and P-tau are risk factors of POD. However, the CSF levels of Aβ_42_, Aβ_42_/T-tau, and Aβ_42_/P-tau are protective factors of POD by univariate analyses. Adding adjustments for age, gender, years of education, and MMSE scores, multivariate logistic regression analyses show results identical to the multivariate logistic regression analyses, as shown in Table 3.1. Sensitivity analyses also present the same results, as shown in Supplementary Table S1.

**Table 3.1:** Logistic analysis for risk factors of POD patients.

	**Unadjusted**		**Adjusted**	
**Factors of interest**	**Odds ratio (95% CI)**	***P*-value**	**Odds ratio (95% CI)**	***P*-value**
MCI	3.387(2.382–4.815)	0.001	1.696(1.035–2.779)	0.036
Preoperative CSFAβ_42_	0.997(0.997–0.998)	0.001	0.998(0.997–0.999)	0.006
Preoperative CSF T-tau	1.006(1.005–1.007)	0.001	1.006(1.004–1.008)	0.001
Preoperative CSF P-tau	1.063(1.052–1.074)	0.001	1.060(1.044–1.075)	0.001
Preoperative CSFAβ_42_/ T-tau	0.393(0.316–0.489)	0.001	0.493(0.376–0.647)	0.001
Preoperative CSFAβ_42_/ P-tau	0.833(0.796–0.872)	0.001	0.867(0.827–0.910)	0.001

Adjusted by gender, age, years of education and MMSE for patients over the age of 50~90.

### Logistic regression analysis of the influencing factors of MCI

With MCI as the dependent variable, logistic regression analyses were carried out to explore the influencing factors. The results show that the CSF levels of P-tau and T-tau are risk factors, and CSF levels of Aβ_42_, Aβ_42_/T-tau, and Aβ_42_/P-tau are protective factors of MCI by univariate analyses. Adding adjustments for age, gender, years of education, and MMSE scores, multivariate logistic regression analyses demonstrate identical results, as shown in Table 3.2. Sensitivity analyses also show the same results, as in Supplementary Table S2. The relationship between MCI and biomarkers in CSF is not affected by gender, as shown in Supplementary Table S3.

**Table 3.2:** Logistic analysis for risk factors of MCI patients.

**Factors of interest**	**Unadjusted**		**Adjusted**	
	**Odds ratio (95% CI)**	***P*-value**	**Odds ratio (95% CI)**	***P*-value**
Preoperative CSFAβ_42_	0.998 (0.997–0.999)	0.001	0.998 (0.997–0.999)	0.001
Preoperative CSF T-tau	1.005 (1.004–1.006)	0.001	1.004 (1.003–1.005)	0.001
Preoperative CSF P-tau	1.051 (1.042–1.061)	0.001	1.046 (1.036–1.056)	0.001
Preoperative CSFAβ_42_/ T-tau	0.572 (0.498–0.657)	0.001	0.609 (0.529–0.702)	0.001
Preoperative CSFAβ_42_/ P-tau	0.873 (0.846–0.900)	0.001	0.885 (0.859–0.912)	0.001

Adjusted by gender, age, years of education and MMSE for patients over the age of 50~90.

### Causal mediation analyses

**Figure 3:**
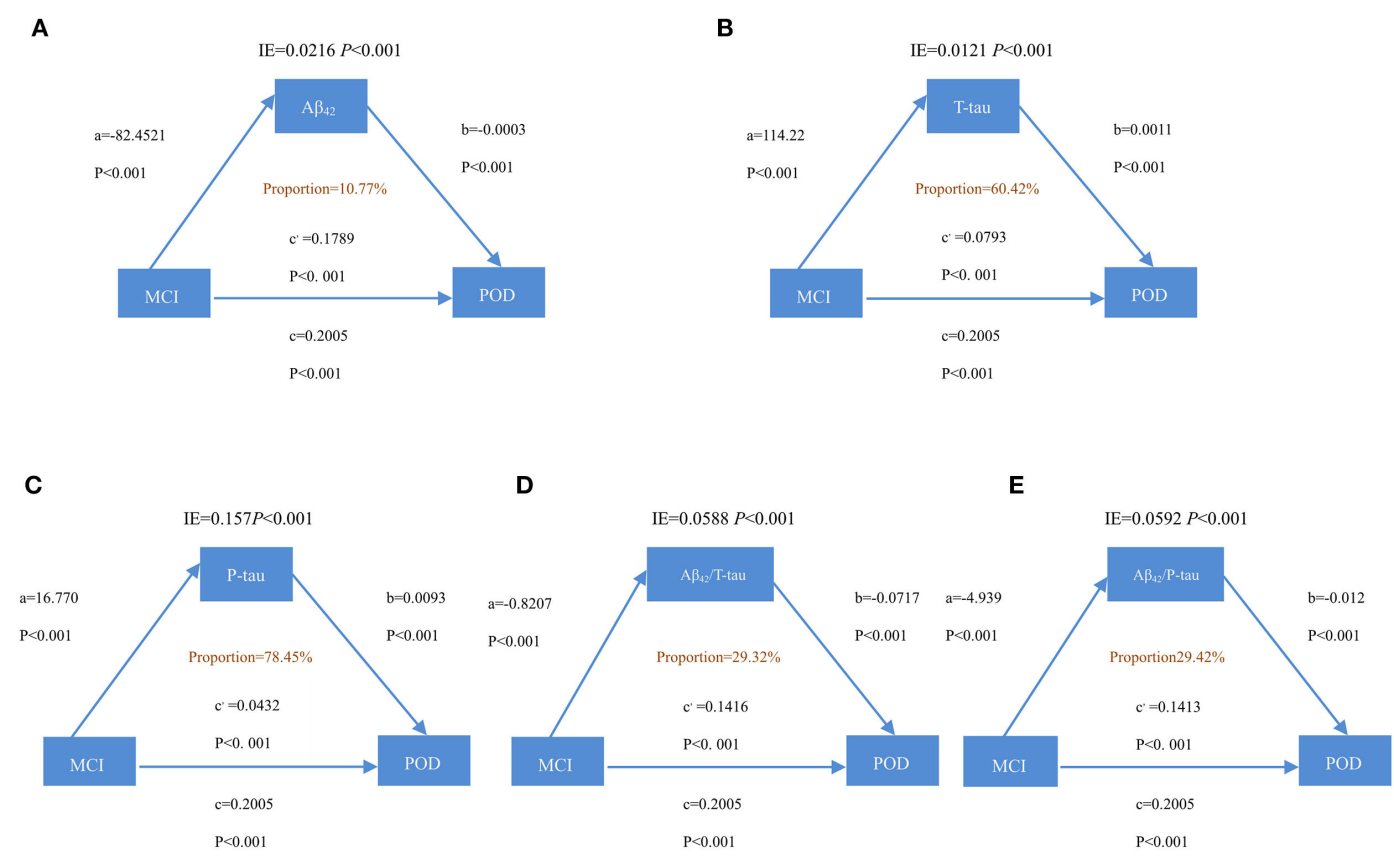
Mediation analyses with POD as a cognitive outcome. In the PNDABLE (Perioperative Neurocognitive Disorder and Biomarker LifestylE study), the relationship between MCI and POD was mediated by amyloid pathology indicated by **(A)** amyloid β 42 (Aβ_42_), **(B)** Total-tau (T-tau), **(C)** phosphorylated total-tau (P-tau), **(D)** amyloid β 42/ Total-tau (Aβ_42_/ T-tau), and **(E)** amyloid β 42/phosphorylated total-tau (Aβ_42_/ P-tau). IE, indirect effect.

It is discovered that the relationship between MCI and POD is mediated by Aβ_42_, T-tau, P-tau Aβ_42_/T-tau, and Aβ_42_/P-tau. This effect is considered as partial mediation, with the mediation proportion varying from 10.77 to 78.45%, as shown in Figure 3.

## Discussion

In the present study, the incidence of POD is 20.2%, which is consistent with the results in a previous study of 3.6–41% (Leung et al., 2013). Several studies have shown that 20% of patients experience POD after total knee and hip replacements under spinal anesthesia (Xie et al., 2014). Based on this study, MCI, CSF T-tau, and CSF P-tau are independently associated with POD. In addition, CSF Aβ_42_, Aβ_42_/T-tau, and Aβ_42_/P-tau all act independently as protective factors of POD. In recent years, researchers have been focusing on finding ideal biological markers that indicate POD so as to reduce the occurrence of POD. The biochemical indicator, Aβ and Tau protein, all play an important role in the pathogenesis of POD (Li et al., 2014). Evidence also suggests that the preoperative Aβ and Tau levels in cerebrospinal fluid are significantly related to the changes of postoperative cognitive dysfunction in elderly patients (Wu et al., 2018), which echoes with the present study. Results are not affected by adding more covariates or including patients of different ages (65–90).

As per the current study, the incidence of POD is 32.5% in the MCI group and 12.4% in the NMCI group. Also, changes in CSF Aβ_42_ and P-Tau may appear ahead of the symptoms of cognitive impairment on patients, and the above-mentioned changes in CSF biomarkers may also exist in patients with MCI (Sperling et al., 2011). Jack, Van et al. (Jack et al., 2013) conducted dynamic observation and found that the level of P-Tau in cerebrospinal fluid increases with the decline of cognitive function. These mentioned studies appear to support the assumption that P-Tau is associated with the severity of cognitive impairment, making it a valuable prognostic indicator. Two previous population-based studies examined risk of death among patients with MCI. Both reported that, in the follow-up period, MCI patients with CSF P-Tau have a mortality rate ~1.7 times to that of those without cognitive impairment (Frisoni et al., 1999). Most studies were small scaled, with fewer than 100 subjects, each with mild cognitive impairment or no cognitive impairment, and did not adjust for variables associated with the development of cognitive dysfunction, e.g., age, gender, and educational background (Morris et al., 2001). To our knowledge, the current study is the first large sample report on the relative risk of POD and MCI. Our findings showed that CSF T-tau and CSF P-tau are independent risk factors of MCI. Moreover, CSF Aβ_42_, Aβ_42_/T-tau, and Aβ_42_/P-tau are independent protective factors of MCI.

The study also discovered, for the first time, that the influence of MCI on POD is partially mediated by amyloid pathology and Tau. Based on the above findings, it could also be conceivably hypothesized that amyloid pathology and Tau could modulate the relationship of MCI and cognition impairment *via* mediation effects (the mediation proportion ranges from 10.77 to 78.45%). Normally, only Aβ can be detected in trace amounts in human brain tissue, yet, under specific pathological conditions, the clearance effect on Aβ by neurons to extracellular is reduced, and accumulated Aβ can stimulate nerve cells and glial cells (Salvadores et al., 2020). Accumulated Aβ also promotes the production of cytokines, which, in turn, transforms Aβ from soluble to insoluble and, eventually, into filaments deposited both intracellularly and extracellularly. Abnormal accumulation of Aβ initiates the cascade reactions within nerve cells, such as synaptic damage, excessive phosphorylation of Tau protein, formation of neurofibrillary tangles, and eventual damage on neurons, resulting in neuronal death and memory and cognitive dysfunction (Plotkin and Cashman, 2020). It has been noticed that accumulated Aβ protein in the brain could be high, while Aβ protein in CSF could be low. The possible mechanism behind this phenomenon remains unclear, and researchers have proposed that Aβ protein in the CSF is accumulated in the corresponding brain region, thereby reducing the amount of Aβ protein in the CSF (Zhang et al., 2018). Under normal physiological conditions, the human brain contains only 2–3 mol of phosphorylated Tau protein (P-Tau), and neuronal P-Tau is at a very low level. In case 3–4 times of P-Tau deposits exist in abnormal neurons around Aβ in the brain, it becomes abnormal phosphorylation of Tau protein (John and Reddy, 2021). Tau protein in normal human brains contains about two phosphoric acid molecules per molecule, but the number of phosphorylation sites of Tau protein in the brain of patients with cognitive impairment is increased, which is prone to induce phosphorylation (Didonna, 2020). Phosphorylated Tau protein does not bind to tubulin but inhibits and destroys the formation of microtubules (Li et al., 2007). Excessive Aβ causes a chain reaction, including increased production of phosphorylated Tau protein, which ultimately leads to decreased cognitive function (Ballatore et al., 2007). It has also been suggested that the aggregation of Aβ requires the presence of a certain microtubule-associated protein Tau (Roberson et al., 2007). To address that, a “two-channel-mechanism hypothesis” has been proposed (Small and Duff, 2008). According to this hypothesis, the same upstream mechanism triggers Aβ and Tau, respectively, and the developments of the two are independent and mutually promoting, and eventually lead to cognitive decline jointly. Studies have found that the content of Aβ_42_ in cerebrospinal fluid decreases with the aggravation of MCI condition, showing a negative correlation between the severity of MCI and Aβ_42_ levels (Jack et al., 2010). Phillip et al. found that P-tau in CSF is significantly associated with right hippocampus atrophy, indicating that P-tau protein may be involved in patients with MCI. The hippocampus is an organ related to human learning and memory, while the most common symptom of MCI is amnesia, which echoes the correlation between P-tau and MCI (Thomann et al., 2009). Therefore, one of the issues that emerge from these findings is that amyloid pathology and Tau may play important roles in the process of MCI-induced POD.

A number of limitations need to be noted regarding the present study. First, the number of the participants was limited. More eligible participants will be included in future studies. Second, this study is single-centered, which could be further validated by multicenter studies to come. Third, this study focused on the relationship between MCI and the biomarkers in patients' CSF with preliminarily discussions on the pathogenesis of POD caused by MCI, which depended on further research to prove the current findings *via* animal experiments and further probe into relevant mechanisms. Considerably, more work will need to be done to consolidate the results of the current study. With societies globally entering the state of population aging, despite the life quality at large of senior citizens has been improved greatly, MCI remains an ever-social issue. A reasonable approach to tackle this issue could be to enhance public knowledge of MCI and promote low cardiovascular and cerebrovascular risk life routines, especially among senior citizen groups. Proactive intervention on cardiovascular and cerebrovascular disease risk factors should also be implemented. Moreover, early detection and intervention are also vital as they can prevent or improve cognitive decline.

## Conclusion

The evidence from this study suggests that MCI may be a sound prognostic factor of POD development. Additionally, in general, amyloid pathology and Tau protein might partially mediate the influence of MCI on POD.

## Data availability statement

The raw data supporting the conclusions of this article will be made available by the authors, without undue reservation.

## Ethics statement

The studies involving human participants were reviewed and approved by the Clinical Trial Ethics Committee of Qingdao Municipal Hospital. The patients/participants provided their written informed consent to participate in this study.

## Author contributions

YB conceived the current study. JW, GZ, and CM performed the experiments. FW and XT analyzed data. XL, RD, and BW performed the experiments and wrote and revised the manuscript. All authors have contributed to the manuscript, revising and editing critically for important intellectual content and given final approval of the version and agreed to be accountable for all the aspects of the work presented here. All authors read and approved the final manuscript.

## Funding

This work was supported by National Natural Science Foundation Youth Project (91849126).

## Conflict of interest

The authors declare that the research was conducted in the absence of any commercial or financial relationships that could be construed as a potential conflict of interest.

## Publisher's note

All claims expressed in this article are solely those of the authors and do not necessarily represent those of their affiliated organizations, or those of the publisher, the editors and the reviewers. Any product that may be evaluated in this article, or claim that may be made by its manufacturer, is not guaranteed or endorsed by the publisher.
