# Supplementary material for: The relationship between mild cognitive impairment and postoperative delirium undergoing total knee arthroplasty: The PNDABLE study
Source: Front Aging Neurosci. 2022 Sep 29;14:959510. doi: 10.3389/fnagi.2022.959510 (PMC9559362; doi:10.3389/fnagi.2022.959510)
Supplement: Supplementary file 1 [file Data_Sheet_1.doc]

| Factors of interest | Unadjusted | | Adjusted | |
| --- | --- | --- | --- | --- |
|  | odds ratio (95% CI) | *P*-value | odds ratio (95% CI) | *P*-value |
| MCI | 3.387(2.382-4.815) | 0.001 | 1.736(1.048-2.876) | 0.032 |
| Preoperative CSFAβ_42_ | 0.997(0.997-0.998) | 0.001 | 0.998(0.997-0.999) | 0.006 |
| Preoperative CSF T-tau | 1.006(1.005-1.007) | 0.001 | 1.006(1.004-1.008) | 0.001 |
| Preoperative CSF P-tau | 1.063(1.052-1.074) | 0.001 | 1.061(1.045-1.077) | 0.001 |
| Preoperative CSFAβ_42_/ T-tau | 0.393(0.316-0.489) | 0.001 | 0.495(0.377-0.652) | 0.001 |
| Preoperative CSFAβ_42_/ P-tau | 0.833(0.796-0.872) | 0.001 | 0.866(0.825-0.908) | 0.001 |

**Table S1. The first sensitivity analysis.**

*In the first sensitivity analysis for risk factors of POD patients adjusted by gender, age, years of education, MMSE, BMI, type 2 diabetes (yes or no), hypertension (yes or no), smoking (yes or no), coronary heart disease (yes or no), and alcohol intake (yes or no) for patients.

**Table S2. The second sensitivity analysis.**

| Factors of interest | Unadjusted | | Adjusted | |
| --- | --- | --- | --- | --- |
|  | odds ratio (95% CI) | *P*-value | odds ratio (95% CI) | *P*-value |
| Preoperative CSFAβ_42_ | 0.998(0.997-0.999) | 0.001 | 0.998(0.997-0.999) | 0.001 |
| Preoperative CSF T-tau | 1.005(1.004-1.006) | 0.001 | 1.004(1.003-1.005) | 0.001 |
| Preoperative CSF P-tau | 1.052(1.042-1.062) | 0.001 | 1.047(1.037-1.057) | 0.001 |
| Preoperative CSFAβ_42_/ T-tau | 0.572(0.498-0.657) | 0.001 | 0.620(0.538-0.715) | 0.001 |
| Preoperative CSFAβ_42_/ P-tau | 0.873(0.847-0.900) | 0.001 | 0.886(0.860-0.913) | 0.001 |

**In the second sensitivity analysis for Risk factors of MCI patients adjusted by gender, age, years of education, MMSE, BMI, type 2 diabetes (yes or no), hypertension (yes or no), smoking (yes or no), coronary heart disease (yes or no), and alcohol intake (yes or no) for patients.

**Table S3 Analysis whether gender affected the relationship of MCI and CSF POD biomarkers.**

|  | Gender | |
| --- | --- | --- |
|  | F | P |
| MCI and CSFAβ_42_ | 0.042 | 0.838 |
| MCI and CSF T-tau | 3.844 | 0.050 |
| MCI and CSF P-tau | 0.186 | 0.666 |
| MCI and CSFAβ_42_/ T-tau | 0.146 | 0.702 |
| MCI and CSFAβ_42_/ P-tau | 0.034 | 0.855 |
